# Soluble CD40 Ligand as a Promising Biomarker in Cancer Diagnosis

**DOI:** 10.3390/cells13151267

**Published:** 2024-07-28

**Authors:** Alireza Pazoki, Sepehr Dadfar, Alireza Shadab, Dariush Haghmorad, Valentyn Oksenych

**Affiliations:** 1 Department of Immunology, School of Medicine, Semnan University of Medical Sciences, Semnan 35147-99442, Iran; 2 Department of Health Science, Iran University of Medical Sciences, Tehran 14496-14535, Iran; 3 Cancer Research Center, Semnan University of Medical Sciences, Semnan 35147-99442, Iran; 4 Broegelmann Research Laboratory, Department of Clinical Science, University of Bergen, 5020 Bergen, Norway

**Keywords:** biomarker, CD40 ligand, inflammation, malignancy, cancer

## Abstract

Cancer remains a significant challenge in medicine due to its complexity and heterogeneity. Biomarkers have emerged as vital tools for cancer research and clinical practice, facilitating early detection, prognosis assessment, and treatment monitoring. Among these, CD40 ligand (CD40L) has gained attention for its role in immune response modulation. Soluble CD40 ligand (sCD40L) has shown promise as a potential biomarker in cancer diagnosis and progression, reflecting interactions between immune cells and the tumor microenvironment. This review explores the intricate relationship between sCD40L and cancer, highlighting its diagnostic and prognostic potential. It discusses biomarker discovery, emphasizing the need for reliable markers in oncology, and elucidates the roles of CD40L in inflammatory responses and interactions with tumor cells. Additionally, it examines sCD40L as a biomarker, detailing its significance across various cancer types and clinical applications. Moreover, the review focuses on therapeutic interventions targeting CD40L in malignancies, providing insights into cellular and gene therapy approaches and recombinant protein-based strategies. The clinical effectiveness of CD40L-targeted therapy is evaluated, underscoring the need for further research to unlock the full potential of this signaling pathway in cancer management.

## 1. Introduction

Cancer remains one of the most pressing challenges in modern medicine, with its complex etiology and heterogeneous nature posing significant obstacles to effective diagnosis and treatment [1]. In this context, biomarkers play a crucial role in cancer research and clinical practice, offering valuable tools for early detection, prognosis assessment, and treatment monitoring [2]. By providing insights into the molecular signatures associated with cancer development and progression, biomarkers facilitate personalized medicine approaches and improve patient outcomes [3].

Among the multitude of biomarkers under investigation, CD40 ligand has emerged as a key player in the intricate network of immune response and inflammation [4]. CD40 ligand, a transmembrane protein primarily expressed on activated T cells, plays a pivotal role in modulating immune cell interactions and orchestrating the adaptive immune response [5]. Through its interactions with CD40 receptors expressed on various cell types, including B cells, dendritic cells, and endothelial cells, CD40 ligand regulates diverse cellular processes, ranging from antigen presentation and antibody production to inflammation and tissue remodeling [6].

The soluble form of CD40 ligand (sCD40L) has garnered increasing attention as a potential biomarker in cancer diagnosis and progression. Shed from the cell surface upon activation, sCD40L enters circulation and reflects the dynamic interplay between immune cells and the tumor microenvironment [7]. Emerging evidence suggests that alterations in sCD40L levels may correlate with cancer development, aggressiveness, and treatment response, thereby holding promise as a non-invasive tool for cancer detection and monitoring [8].

This article aims to elucidate the intricate relationship between sCD40L and cancer, shedding light on its diagnostic and prognostic potential. By synthesizing existing knowledge and highlighting recent advancements, we seek to unravel the multifaceted role of sCD40L in cancer biology. Through comprehensive analysis and critical appraisal, our objective is to provide insights that may inform future research endeavors and pave the way for innovative strategies in cancer management.

## 2. Biomarker Discovery and Significance

Biomarkers have been integral to cancer research and clinical practice, serving as measurable indicators of biological processes, disease progression, or treatment response [2,9]. The historical trajectory of biomarker discovery in cancer research reflects a continuous quest for more accurate, reliable, and clinically relevant markers to improve diagnostic capabilities and treatment outcomes [10]. In the early stages of cancer research, biomarker discovery primarily relied on observable clinical manifestations and gross anatomical changes [11]. However, advancements in molecular biology and technology have revolutionized the field, enabling the identification and characterization of biomarkers at the cellular and molecular levels [12]. From single gene mutations to complex molecular signatures, biomarkers have evolved to encompass a wide range of molecular, genetic, proteomic, and metabolomics entities [13].

The importance of identifying reliable biomarkers for early detection and monitoring of cancer cannot be overstated. Early detection significantly improves patient prognosis by enabling timely intervention and treatment initiation when the disease is most amenable to curative therapies [14,15]. Furthermore, biomarkers play a crucial role in monitoring disease progression, assessing treatment response, and predicting patient outcomes, thereby guiding clinical decision-making and personalized treatment strategies [16].

Soluble biomarkers, including proteins, nucleic acids, and metabolites, hold particular promise in cancer research and clinical practice. Unlike tissue-based biomarkers, soluble biomarkers can be easily sampled from accessible body fluids such as blood, urine, or saliva, offering non-invasive and minimally invasive approaches to cancer detection and monitoring [9,17]. Furthermore, advances in detection methods, such as immunoassays, mass spectrometry, and next-generation sequencing, have enhanced the sensitivity and specificity of soluble biomarker detection, enabling high-throughput analysis and multiplexed profiling [18].

Despite the growing repertoire of biomarkers in oncology, existing markers often exhibit limitations in terms of sensitivity, specificity, and clinical utility. Many biomarkers lack the requisite accuracy to distinguish between benign and malignant conditions or to predict disease outcomes reliably [19,20]. Moreover, biomarkers may exhibit inter-individual variability, temporal fluctuations, and non-specific associations with other physiological or pathological processes, complicating their interpretation and clinical application [21].

This inherent heterogeneity and variability underscore the need for novel and reliable biomarkers like soluble CD40 ligand (sCD40L) in cancer research and clinical practice. By overcoming the limitations of existing markers and offering unique insights into the tumor-immune interface, sCD40L holds promise as a valuable tool for cancer diagnosis, prognosis, and treatment stratification. As such, continued exploration of sCD40L and other emerging biomarkers is essential to advance our understanding of cancer biology and improve patient outcomes in oncology [22,23].

## 3. CD40 Ligand and Cancer

The CD40 ligand (CD40L), or CD154, is an essential protein in the immune system. It belongs to the TNF superfamily [24], which is a type II transmembrane protein. It is located on the X chromosome at Xq26.3 and is primarily expressed in activated T cells, particularly in a specific subset called T follicular helper cells (TFH cells) [25]. CD40L plays a crucial role in regulating immune responses and inflammatory processes. The impact of CD40L in different cancers varies depending on the cancer type and other factors such as the patient’s immune response [26]. CD40L plays a crucial role in activating immune cells and has the potential to influence the tumor microenvironment, impacting cancer progression and treatment effectiveness [27].

CD40L is crucial in promoting the interaction between T cells and B cells [28]. Recent research has highlighted that T cells can transfer CD40L to antigen-presenting B cells through a mechanism that depends on both contacts and the presence of antigens [29]. This transfer is essential for providing “help” to B cells, which is necessary for the production of high-affinity antibodies. The interplay between CD40 on B cells and CD40L on T cells triggers B cell activation, proliferation, and immunoglobulin class switching, all indispensable for arranging an effective immune response [30].

The interaction between CD40 and CD40L significantly impacts immune regulation, especially concerning APCs. This interaction directly engages B cells and CD4+ T cells while also impacting CD8+ T cells. A recent study has revealed that CD40L stimulation not only enhances the numbers of peripheral CD8+ T cells in humans but also contributes to the generation of memory CD8+ T cells [31]. Furthermore, CD40L plays an important role in activating APCs, including dendritic cells, which initiates adaptive immune responses necessary for the immune system’s recognition and elimination of tumor cells [32].

## 4. CD40 Roles in Inflammatory Responses

CD40L has diverse roles in inflammatory responses, including the induction of pro-inflammatory cytokines production such as IL-1, IL-6, and TNF-α [33]. This leads to the activation of endothelial cells, recruitment of immune cells, and acute phase reactions [34]. Regarding endothelial cells, CD40L from platelets triggers an inflammatory response by inducing the expression of adhesion molecules, cytokines, chemokines, and inflammatory mediators important for wound healing and atherosclerosis [35]. This facilitates leukocyte recruitment and may contribute to inflammatory diseases [36]. CD40L is also involved in inflammation-driven angiogenesis, where it upregulates pro-angiogenic factors like vascular endothelial growth factor (VEGF) [37]. This may have implications for tumor growth and metastasis [24].

The interaction between CD40 and CD40L enhances immune and inflammatory responses against cancer cells. It activates intracellular signaling pathways such as phosphoinositide 3-kinases (PI3K)/AKT, mitogen-activated protein kinases (MAPKs), and Janus kinase 3 (JAK3)/signal transducer and activator of transcription proteins (STATs), leading to immunogenic cell death, antigen presentation, and upregulation of major MHC molecules [38,39,40,41,42,43]. CD40L also stimulates the production of pro-inflammatory cytokines, including IL-12, which promotes anti-tumor effects [44]. Overall, the CD40–CD40L interaction has a multifaceted role in anti-tumor immunity and presents a promising target for cancer immunotherapy. The following sections explain all the details.

CD40L can stimulate the generation of pro-inflammatory cytokines and chemokine, which possess the capability to attract and activate immune cells within the tumor microenvironment. The production of these pro-inflammatory factors by CD40L not only draws immune cells to the tumor site but also enhances their anti-tumor activity [45,46].

## 5. CD40 Interaction with Tumor Cells

CD40L can activate APCs like dendritic cells and macrophages. This activation process improves the presentation of antigens, which is a critical step in the activation of T cells. APCs play a central role in the immune response by presenting antigens to T cells. This activation of APCs by CD40L boosts the immune system’s ability to identify tumor-specific antigens and initiate a targeted immune response against cancer cells [27]. Additionally, CD40L can enhance the upregulation of MHC molecules in tumor cells. The increased expression of MHC molecules facilitated by CD40L improves the detectability of cancer cells by the immune system [47].

Immunogenic Cell Death (ICD) is a specific type of cell death that is different from the normal process that goes unnoticed by the immune system. In ICD, the immune system is activated to respond against the antigens of the dying cells [48]. When tumor cells undergo ICD, they release specific molecules that stimulate an immune response against the tumor. CD40L facilitates this phenomenon in the context of cancer by helping the immune system recognize and actively fight against the cancer cells. In summary, CD40L plays a pivotal role in boosting the immune response to tumor cells undergoing ICD [49]. This process enables the immune system to identify and combat cancer cells more effectively [50].

**Figure 1:**
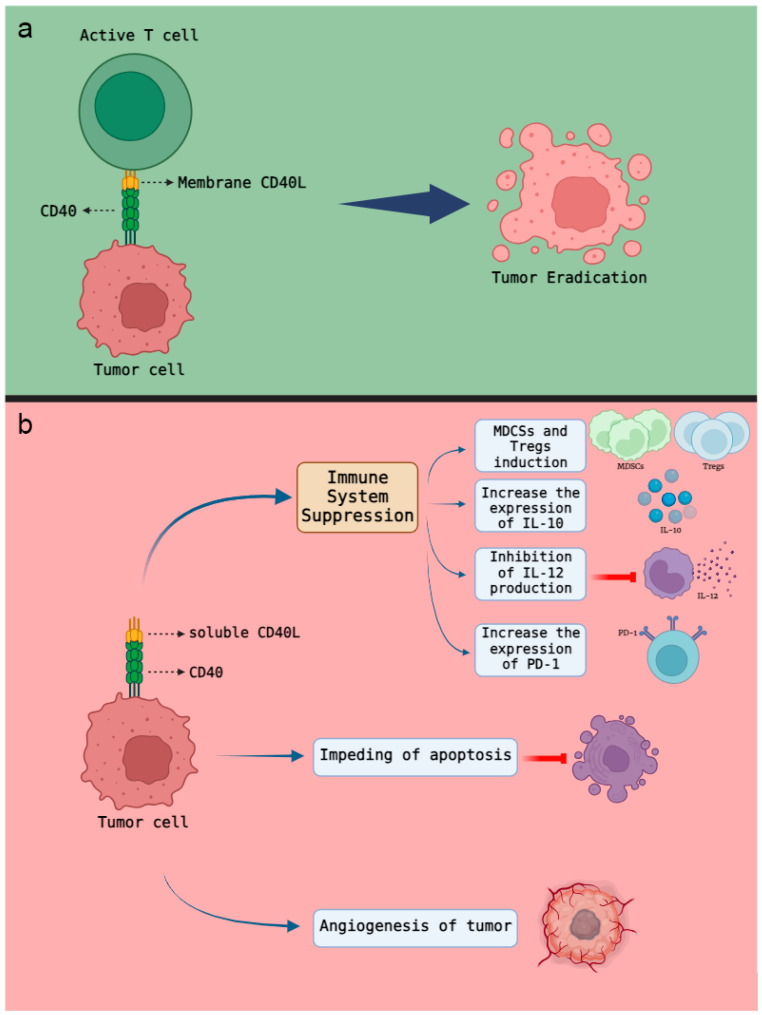
The impact of different forms of CD40L on tumor cells. (**a**) Binding of membrane CD40L and CD40 can directly enhance the process of tumorcell apoptosis. (**b**) The soluble CD40L has limited signaling potency when interacts with CD40 on tumor cells. As a result, this interaction supports tumor survival by hindering apoptosis, dampening the immune system, and promoting tumor angiogenesis. (MDSCs: Myeloid-derived suppressor cells; PD-1: programmed cell death-1; Tregs: Regulatory T cells).

CD40L can increase the susceptibility of tumor cells to lysis, or destruction, by T cells (Figure 1a). This suggests that the immune system can more efficiently eliminate tumor cells [27]. CD40L has the potential to regulate Tumor-Associated Macrophages (TAMs), shifting them from a pro-tumor (M2) to an anti-tumor (M1) phenotype, which can reduce tumor growth and spread [51].

The effects of CD40L on different types of cancer are summarized in Table 1. However, it is important to note that the roles of CD40L can vary depending on the specific type, location, and expression levels of CD40L and CD40 within different tumor tissues [52].

## 6. sCD40L as a Biomarker

Following our exploration of the role of CD40L in cancer, we will now turn our attention to the potential of sCD40L as a biomarker in diverse cancer types. By investigating the diagnostic and prognostic value of sCD40L, we aim to shed light on its significance in cancer detection, prognosis, and treatment response assessment. Soluble CD40 ligand, an 18 kDa protein and a member of the TNF superfamily, is primarily released from the surface of T-lymphocytes and activated platelets [55,65]. The majority (over 95%) of sCD40L is released from activated platelets through an enzymatic cleavage at a metalloproteinase-sensitive site located in the membrane-proximal region of its extracellular domain [66,67].

sCD40L can exist as either a monomer or a trimer. The monomeric form is a single molecule of the protein generated by cleavage of the membrane-bound CD40 ligand. The trimeric form consists of three CD40 ligand molecules held together by non-covalent interactions and is considered the biologically active form. The relative abundance of monomeric and trimeric sCD40L can vary, and their specific contributions to function are still under active research [55,68].

Measuring serum sCD40L levels in research involves several methods, with enzyme-linked immunosorbent assay (ELISA) being one of the most commonly used [22,55,67]. The ELISA method enables the precise measurement of sCD40L levels in serum samples, offering an accurate method to evaluate its concentration. Additional techniques include multiplex bead-based assays [69] and Luminex technology [70], which enable the simultaneous quantification of several cytokines and soluble factors, including sCD40L.

As mentioned, CD40/CD40L ligation plays a dual role in tumor biology. Membrane-bound CD40L exhibits potent signaling capabilities upon binding to CD40 on tumor cell surfaces, enabling tumor eradication. Conversely, the soluble form of CD40L found in serum possesses limited signaling strength when interacting with CD40 on tumor cells. Consequently, this interaction promotes tumor survival by impeding apoptosis, suppressing the immune system, and promoting tumor angiogenesis (Figure 1b) [55,71].

sCD40L utilizes various mechanisms to suppress the immune system. Firstly, it induces the generation of Myeloid-derived suppressor cells (MDSCs) and T regulatory cells while increasing the expression of interleukin 10. These actions inhibit antigen presentation, cytokine production, macrophage activation, and antigen-specific T-cell proliferation. Additionally, sCD40L inhibits the production of IL-12 from activated monocytes. Moreover, it upregulates the expression of programmed cell death-1 (PD-1), a crucial regulator of T-cell exhaustion [55,72]. In the tumor microenvironment, sCD40L, particularly when present at high levels (e.g., in ovarian cyst fluid), exerts greater immune inhibitory effects [73]. Its role in apoptosis inhibition stems from its interference with the downstream signaling of Fas and Fas ligands, which possess an intra-cellular ‘death domain’ capable of triggering apoptosis. Finally, its ability to promote angiogenesis is supported by its association with hepatocyte growth factor (HGF) and vascular endothelial growth factor (VEGF) [74].

sCD40L serves as a valuable biomarker in various cancers. Generally, this biomarker provides insights into the disease prognosis, independent of tumor size and lymph node involvement [66,67]. Remarkably, common cancer treatments such as surgery, chemotherapy, and radiotherapy have been observed to decrease the plasma and serum levels of sCD40L [66]. Research has measured the serum or plasma levels of sCD40L in diverse cancer types, with notable increases reported predominantly. Noteworthy examples of cancers include lung [75], nasopharynx [66], ovary [73], prostate [55], and malignant bone tumors [76], among others. A summarized compilation of these findings can be found in Table 2.

One of the advantages of using sCD40L as a biomarker is that its level remains consistent regardless of the patient’s age or sex [79]. sCD40L is an indicator of the prognosis and response to cancer treatment [66,67]. However, there are some challenges associated with using this biomarker. Firstly, its level increases in inflammatory conditions and other cancers, which makes it unsuitable as a standalone biomarker [79]. Secondly, it is unclear whether the release of sCD40L in the blood circulation is caused by cancer or simply the activation of platelets in these patients [65]. Therefore, more studies are needed to establish the roles of sCD40L and understand its mechanism in all types of cancers.

## 7. Clinical Applications and Future Directions

Because of the pivotal role CD40 plays in anti-tumor immune responses, diverse strategies have been investigated to activate CD40 signaling. These strategies can be broadly categorized into two groups: those relying on agonistic antibodies and those based on CD40L. The CD40L-based approaches within this category can be subdivided into 1-recombinant protein methods utilizing CD40L mimetics and 2-gene therapy approaches, which involve introducing the CD40L gene into target cells; when CD40L enters the body, it cannot specifically distinguish tumor tissue from normal tissue. Therefore, a common strategy is to inject CD40L directly at the tumor site. Doing so aims to enhance the immune response specifically within the tumor microenvironment, as highlighted in clinical trial NCT03225989. Often, these injections are administered concurrently with cytokines like IL-2, which further amplify the immune activation —an approach also documented in clinical trial NCT00458679.

Unlike agonist therapy, CD40/CD40L antagonist therapy disrupts the CD40/CD40L interaction and suppresses immune responses by impeding CD40 signaling and deactivating immune cells. This approach proves advantageous in the context of autoimmune diseases and allograft transplantation.

As agonistic antibodies and CD40/CD40L antagonist approaches fall outside the focus of this review, this section will specifically address therapeutic interventions directed at CD40L in malignancies (Table 3).

## 8. Cellular and Gene Therapy Based on CD40L

The initial strategies focused on targeting CD40 involved manipulating the increased expression of CD40L in diverse cell groups. Indeed, this can be categorized into two primary methods: cellular vaccines expressing CD40L and CD40L-specific gene therapy employing delivery platforms like oncolytic viruses. Both approaches rely on the natural ligand (CD40L) to trigger CD40 signaling. Pre-clinical evidence suggests that CD40L or CD40 antibodies can be used to induce DC and macrophage-mediated cytokine release, antigen processing, tumor stroma destruction, and T-cell activation against established malignancies [82].

A tumor-specific immune response can be elicited by co-expressing CD40L with other immunostimulatory molecules through either plasmid transfection or mRNA electroporation in bystander cells or autologous dendritic cells. For instance, the co-expression of CD40L with GM-CSF in bystander cells and with toll-like receptor 4 (TLR4)/CD70 or TLR4/CD70/tumor antigen in autologous DCs was employed for the treatment of advanced lung adenocarcinoma and advanced melanoma. During phase 1 to 2 trials, these combinations facilitated the infiltration of lymphocytes into tumors [83,84,85,86].

During a phase 1 clinical trial involving patients with chronic lymphocytic leukemia (CLL), the infusion of autologous CLL cells transduced with adenovirus encoding murine mCD40L resulted in the generation of tumor-specific T cells and antibody responses, leading to tumor remission [87]. Nevertheless, a subset of patients developed antibodies against murine CD40L. To address this issue, the researchers subsequently conducted another phase 1 trial using CLL cells transduced with adenovirus encoding ISF35 (humanized mCD40L). The CLL cells modified with ISF35 exhibited anti-tumor activity in patients with CLL. The toxicities associated with ISF35 were primarily mild to moderate flu-like symptoms, including fever, joint pain, muscle pain, nausea, vomiting, and fatigue. These symptoms often appeared a few hours after the infusion and lasted 2–4 days. However, they resolved completely in all cases. There were no dose-limiting toxicities at any dose level [88].

In two studies involving patients with metastatic melanoma, the administration of adenovirus carrying the CD40L gene (Ad-CD40L) triggered T-cell activation, which was associated with extended survival. Similarly, in a trial with individuals diagnosed with bladder cancer, Ad-CD40L facilitated T-cell infiltration into the bladder and exhibited anti-tumor effects. The treatment was generally well received, with minimal adverse reactions. The primary side effect noted was a temporary discomfort experienced during the prewash phase. Postoperatively, urinary tract infections and one case of late septicemia with elevated potassium were reported. It is noteworthy that none of the adverse events were directly attributed to the vector therapy [89,90,91].

More recently, Chimeric Antigen Receptor (CAR) T cells expressing CD40L exhibited greater anti-tumor efficacy in preclinical models. This strategy integrates the target specificity of CAR T cells with CD40L-driven costimulation, creating a “cellular bispecific” approach [92].

## 9. Recombinant Proteins Based on CD40L

These substances act as molecular counterparts to the natural CD40L, engaging exclusively and specifically with CD40 through the receptor-binding domain. CD40 must be correctly trimerized to transmit a productive signal. Thus, there are substances such as purified CD40L, stabilized trivalent/trimeric forms of CD40L, and, more recently, hexavalent/hexameric forms of CD40L. Even though purified CD40L trimerizes in solution, CD40L approaches have not been very successful. However, in combination with IL-6 blockage, stable trimeric forms, such as the isoleucine zipper CD40L variants, have demonstrated some action [93].

Avrend is a trimeric recombinant protein of human sCD40L. In preclinical investigations, it induced a T-cell response and improved antigen presentation in APCs, which stimulated anti-tumor immune responses and resulted in tumor remission in human breast cancer and lymphoma-bearing immune-deficient SCID mice [94,95]. In a phase 1 clinical trial involving individuals with advanced solid tumors or non-Hodgkin lymphoma (NHL), Avrend exhibited anti-tumor efficacy [96].

Recent findings indicate that conveying signals through the TNF-R-SF is more effective with higher-order hexavalent methods [97,98,99,100]. The hexavalent agonists are a class of co-stimulatory TNF-SF ligands that bind to their cognate receptors on target cells and cause clustering of six receptor chains in a spatially specified way. HERA-CD40L and MEDI5083 are fusion proteins composed of a trivalent but single-chain CD40L-receptor-binding domain (scCD40LRBD) coupled to a human IgG, resulting in a hexavalent molecule [98].

HERA-CD40L is formulated with an IgG1 featuring silenced Fc, facilitating effective receptor agonism on CD40-expressing cells without the need for FcγR-mediated cross-linking. HERA-CD40L exhibited significant effects on both B cells and monocytes, influencing NF-κB signaling and macrophage polarization. This substance induced robust NF-κB signaling in treated B cells. Additionally, when monocytes were exposed to HERA-CD40L, it not only facilitated their differentiation towards the M1 spectrum but also triggered the repolarization of M2 spectrum macrophages towards the M1 spectrum phenotype. HERA-CD40L, when applied to in vitro co-cultures of T and B cells, led to potent activation of T cells against tumors, relying on direct interaction with B cells. In vivo experiments using a murine surrogate of HERA-CD40L demonstrated the stimulation of clonal expansion in antigen-specific T cells. Furthermore, as a single agent, it exhibited anti-tumor activity in a colorectal cancer mouse model (CD40-negative syngeneic MC38-CEA). Regarding toxicity, HERA-CD40L is reported to be unaffected by dose-limiting toxicities seen with anti-CD40 antibodies. However, it is crucial to remember that, while these findings are encouraging, they are based on preclinical research. Clinical trials would be required to confirm the safety and toxicity of HERA-CD40L in humans [98].

While recombinant CD40L was one of the initial methods employed for CD40 targeting, its potential is now emerging as a result of integrating insights into the significance of trimerization and three-dimensional arrangement with more advanced CD40L-based compounds. These compounds, being true agonists that do not rely on FcγR-mediated crosslinking, are expected to possess a safer profile compared to alternative approaches.

## 10. Overview of the Clinical Effectiveness of CD40L-Targeted Therapy

Despite the exploration of diverse methods to trigger CD40 signaling, the minimal clinical success over the past two decades implies a necessity to investigate novel approaches. To date, the evaluation of CD40/CD40L-targeted therapy has been limited to early-phase clinical trials involving small groups of patients. The clinical effectiveness of CD40/CD40L anti-tumor therapy seems to be modest, displaying total response rates between 2% and 20% and partial response rates ranging from 4% to 28.6%. In contrast, the outlook for CD40/CD40L anti-autoimmune disease therapy appears encouraging, with clinical response rates ranging from 46% to 75%.

Moreover, recent preclinical investigations have demonstrated heightened potential for tumor control when CD40 agonists are administered in conjunction with additional immunostimulatory therapies. These include the Flt3 ligand, which promotes dendritic cell maturation and survival [101], along with anti-VEGFA/Ang2 monoclonal antibodies that induce tumor microvasculature regression and antigen-presenting cell activation [102]. Additionally, combining CD40 agonists with anti-PD-1 and anti-CTLA4 monoclonal antibodies has shown synergistic effects, enhancing anti-tumor responses [103]. While it is undeniable that CD40/CD40L plays a crucial role in initiating anti-tumor immune responses, the full potential of this signaling pathway has yet to be fully realized by the agonists currently in clinical development.

## 11. Conclusions

The multifaceted role of CD40 ligand (CD40L) and its soluble form (sCD40L) in cancer biology underscores their significance as potential biomarkers and therapeutic targets. The intricate interplay between sCD40L and the tumor microenvironment offers valuable insights into cancer diagnosis, prognosis, and treatment response assessment. While sCD40L shows promise as a non-invasive biomarker across diverse cancer types, its clinical utility necessitates further validation and standardization. Additionally, therapeutic interventions targeting CD40L hold potential for enhancing anti-tumor immune responses, though challenges remain in achieving optimal clinical effectiveness. Continued research efforts are essential to elucidate the full spectrum of CD40L-mediated immune modulation in cancer and to translate these findings into innovative strategies for personalized cancer management. By leveraging the diagnostic and therapeutic potential of CD40L, we can advance towards more effective cancer detection, treatment, and ultimately, improved patient outcomes in oncology.

## Figures and Tables

**Table 1:** The effects of CD40L on different types of cancers.

Cancer Type	Role of CD40L	Immune Cells Involved	Treatment Sensitization	References
Breast cancer	- Activation of dendritic cells and macrophages in the tumor microenvironment.	Dendritic cells, Macrophages	…	[53,54]
	- Induction of IL-12 secretion for T cell and NK cell-mediated antitumor effects.			
	- Induction of apoptosis in cancer cells expressing CD40.			
Lung cancer	- Enhancement of T-cell and NK-cell infiltration into the tumor microenvironment.	T cells, NK cells, Macrophages	Chemotherapy, Radiotherapy	[54,55]
	- Influence on macrophage polarization from pro-tumor (M2) to anti-tumor (M1) phenotype.			
	- Sensitization of cancer cells to chemotherapy and radiotherapy.			
Prostate cancer	- Stimulation of pro-inflammatory cytokine and chemokine production by cancer cells.	Immune cells, Macrophages	…	[56]
	- Attraction and activation of immune cells within the tumor microenvironment.			
	- Reversal of macrophage balance from pro-tumor (M2) to M1 phenotype.			
Colorectal cancer	- Induction of dendritic cell maturation and activation.	Dendritic cells, T cells	…	[54,57]
	- Enhance the presentation of tumor antigens to T cells for a tumor-specific immune response.			
	- Inhibition of colorectal cancer cell growth and angiogenesis through thrombospondin-1 induction.			
Melanoma	- Amplification of T-cell and NK-cell cytotoxicity and proliferation against melanoma cells.	T cells, NK cells	…	[54,58]
	- Stimulation of Fas expression on melanoma cells, leading to apoptosis through FasL interaction.			
Bladder cancer	- Promotion of T-cell and NK-cell infiltration into the tumor microenvironment.	T cells, NK cells, Macrophages	Chemotherapy, Radiotherapy	[54,59]
	- Influence on macrophage polarization from pro-tumor to anti-tumor phenotype			
	- Increase in susceptibility of bladder cancer cells to chemotherapy and radiotherapy			
Non-Hodgkin lymphoma	- Triggering of dendritic cell maturation and activation.	Dendritic cells, T cells	…	[54,60]
	- Enhance the presentation of tumor antigens to T cells for a tumor-specific immune response.			
	- Inhibition of non-Hodgkin lymphoma cell growth and angiogenesis through thrombospondin-1 induction.			
Leukemia	- Promotion of pro-inflammatory cytokine and chemokine production by leukemia cells.	Immune cells, Macrophages	…	[61,62]
	- Activation of immune cells within the tumor.			
	- Shift of macrophage balance from M2 to M1 phenotype.			
Pancreatic cancer	- Activation of dendritic cells and macrophages in the tumor microenvironment.	Dendritic cells, Macrophages	…	[63]
	- Induction of IL-12 secretion for T cell and NK cell-mediated anti-tumor effects.			
	- Induction of apoptosis in pancreatic cancer cells expressing CD40.			
Liver cancer	- Enhancement of T-cell and NK-cell infiltration into the tumor microenvironment.	T cells, NK cells, Macrophages	Chemotherapy, Radiotherapy	[63]
	- Influence on macrophage polarization from M2 to M1 phenotype.			
	- Sensitization of liver cancer cells to chemotherapy and radiotherapy.			
Stomach cancer	- Stimulation of dendritic cell maturation and activation.	Dendritic cells, T cells	…	[63,64]
	- Enhance the presentation of tumor antigens to T cells for a tumor-specific immune response.			
	- Inhibition of stomach cancer cell growth and angiogenesis through thrombospondin-1 induction.			
Ovarian cancer	- Amplification of T-cell and NK-cell cytotoxicity and proliferation against ovarian cancer cells.	T cells, NK cells	…	[59,63]
	- Stimulation of Fas expression on ovarian cancer cells, leading to apoptosis through FasL interaction.			
Cervical cancer	- Enhancement of pro-inflammatory cytokine and chemokine production within cervical cancer cells.	Immune cells, Macrophages	…	[58]
	- Activation of immune cells within the tumor.			
	- Shift of macrophage balance from M2 to M1 phenotype.			

IL-12: Interleukin-12; NK cells: Natural Killer cells; FasL: Fas ligand.

**Table 2:** Summary of investigations on the level of sCD40L in different types of cancers.

Cancer Type	sCD40L Level	Measured in	Note	References
Prostate cancer	Serum levels N/A (Elevated)	Serum	No significant difference was observed in pre- and post-treatment outcomes of the PSA targeting vaccine.	[55]
Breast cancer	Serum levels N/A (Elevated)	Serum		[55]
Gastric cancer	Patients: 3.57 ± 1.63 ng/mL Standards: 1.94 ± 0.86 ng/mL *p* value < 0.01 (Elevated) a	Serum	sCD40L inhibits cancer cell growth and apoptosis.	[65,77]
Malignant bone tumors	Patients: 1.9 ± 1.7 ng/mL Standards: 0.037 ± 0.04 ng/mL *p* value < 0.05 (Elevated) ^a^	Serum		[76]
Hepatitis C virus-related hepatocellular carcinoma	Patients: 9462 ± 2385 pg/mL Standards: 3280 ± 938 pg/mL *p* value < 0.001 (Elevated) ^a^	Serum	- High sensitivity and specificity of sCD40L was observed compared to AFP.	[78]
			- sCD40L is a valuable diagnostic tool in the early diagnosis and screening of patients.	
Multiple myeloma	Patients: 710.8 pg/mL Standards: N/A *p* value < 0.001 (Elevated) ^b^	Serum	- Significant differences were found among disease stages.	[74]
			- There are positive correlations between sCD40L and HGF, VEGF, IL-6, and Ki-67 PI.	
Nasopharyngeal carcinoma	Patients: 15.2 ± 6.4 ng/mL Standards: 6.3 ± 3.6 ng/mL *p* value < 0.001 (Elevated) ^a^	Serum	sCD40L levels may be useful in identifying UNPC patients with occult distant metastases.	[66]
Ovarian cancer	Patients: 0.056 ± 0.043 ng/mL Standards: 0.21 ± 0.008 ng/mL *p* value < 0.001 (Elevated) ^a^	Serum Ovarian cyst fluid	- Impaired apoptosis association with sCD40L. - sCD40L is a potential tool in the monitoring of inflammation.	[73]
			- High concentrations of it in cyst fluid show local immunosuppression.	
Pancreatic ductal adenocarcinoma	Patients: 30,044.2 ± 9747.9 ng/mL Standards: 9170.5 ± 5449.8 ng/mL *p* value < 0.001 (Elevated) ^a^	Serum	Serum sCD40L is correlated with immunosuppression and angiogenesis in PDAC carcinogenesis/progression and is a promising diagnostic and prognostic biomarker for PDAC superior to CA19-9 and CEA.	[79]
Lung cancer	Patients: 0.46 (0.18–0.96) ng/mL Standards: 0.13 (0.05–0.44) ng/mL *p* value < 0.001 (Elevated) ^c^	Plasma	sCD40L levels were significantly higher in squamous cancer compared with adenocarcinoma.	[75]
Chronic lymphocytic leukemia	Patients: 0.80 ng/mL Standard: 0.29 ng/mL *p* value < 0.001(Elevated) ^b^	Serum	sCD40L inhibits apoptosis by interfering with the downstream signaling of Fas and its ligands.	[71]
Colorectal cancer	Plasma levels N/A (Elevated)	Plasma	The level of sCD40L in the plasma is linked to disease progression and the development of distant metastases.	[67,80]
Older people with gastrointestinal tract cancer	Plasma level N/A (Reduced)	Plasma	…	[81]

N/A: not available, PSA: prostate-specific antigen, AFP: alpha-fetoprotein, HGF: hepatocyte growth factor, VEGF: vascular endothelial cell growth factor, IL-6: Interleukin-6, KI-67 PI: Ki-67 proliferation index, UNPC: Undifferentiated nasopharyngeal carcinoma, PDAC: Pancreatic ductal adenocarcinoma, CEA: carcinoembryonic antigen. ^a^ sCD40L levels are presented as mean ± standard deviation. ^b^ sCD40L levels are presented as median. ^c^ sCD40L levels are presented as median (interquartile range).

**Table 3:** Summary of clinical trials based on CD40 ligand in different cancers.

Drug/Intervention	Brief Summary	Phase	Condition	Study Registration Date	Study Record Updates (Last Verified)	Enrollment (Actual)	NCT
CD40 ligand expressing MSLN-CAR T cells	CD40 Ligand Expressing MSLN-CAR T Cell Therapy in MSLN Positive Advanced/Metastatic Solid Tumors	Phase 1 Phase 2	Advanced or Metastatic Solid Tumors	20 January 2023	May 2024	30	NCT05693844
Autologous B-CLL vaccine expressing CD40L and IL2	Treatment of B-Chronic Lymphocytic Leukemia (B-CLL) with Autologous CD40 Ligand and IL-2-Expressing Tumor Cells	Phase 1	Chronic Lymphocytic Leukemia (CLL)	December 2006	1 January 2014	15	NCT00458679
Autologous IL2 and CD40 Ligand-Expressing Tumor Cells + Lenalidomide	Treatment of B-CLL with Autologous IL2 and CD40 Ligand-Expressing Tumor Cells + Lenalidomide	Phase 1	Chronic Lymphocytic Leukemia (CLL)	February 2013	February 2016	15	NCT01604031
Combination of Flt3L And CD40L	Treatment Of Patients with Metastatic Melanoma and Renal Cancer with a Combination of Flt3L and CD40L	Phase 1	Kidney Cancer Melanoma	March 2001	April 2002	unknown	NCT00020540
Recombinant CD40-ligand	Vaccination of HLA-A1 and/or -A2+ Stage III or IV Melanoma Patients with Tumor Peptide—Loaded Autologous Dendritic Cells That Are Generated in the Absence or Presence of CD40 Ligand	Phase 1 Phase 2	Melanoma	October 2002	May 2015	62	NCT00053391
MEM-288 Intratumoral Injection	Intratumoral injection of MEM-288, conditionally replicative oncolytic adenovirus vector encoding transgenes for human interferon beta (IFNβ) and a recombinant chimeric form of CD40-ligand (MEM40) plus Nivolumab	Phase 1	Solid Tumors	23 December 1996	4 September 2014	61	NCT00001564
MEDI5083	MEDI5083 is a novel fusion protein containing a hexameric recombinant human CD40L structure covalently linked to human IgG4p Fc	Phase 1	Advanced Solid Tumors	21 March 2017	July 2020	204	NCT03089645
GM.CD40L	GM-CSF-Producing and CD40L-Expressing Bystander Cell Line (GM.CD40L) Vaccine in Combination with CCL21 for Patients with Stage IV Adenocarcinoma of the Lung	Phase 1 Phase 2	Lung Cancer Adenocarcinoma	26 March 2012	August 2019	73	NCT01433172
LOAd703	Oncolytic adenovirus serotype 5/35 encoding TMZ-CD40L and 4-1BBL	Phase 1 Phase 2	Pancreatic Adenocarcinoma Ovarian Cancer Biliary Carcinoma Colorectal Cancer	1 March 2018	January 2024	50	NCT03225989
Avrend	Trimeric soluble human CD40L	Phase 1	Advanced solid tumors NHL	unknown	unknown	unknown	unknown
Ad-CD40L	Adenovirus carrying human CD40L gene	Phase 1 Phase 2	Malignant Melanoma	September 2011	February 2016	30	NCT01455259
Ad-CD40L-CLL cells	Autologous CLL B Cells Transduced to Express Chimeric CD154 (ISF35)	Phase 1	chronic Lymphocytic Leukemia	June 2006	October 2008	9	NCT00779883
TriMixDC-MEL	A Phase II Trial Using a Universal GM-CSF-Producing and CD40L-Expressing Bystander Cell Line (GM.CD40L) in the Formulation of Autologous Tumor Cell-Based Vaccines for Patients with Malignant Melanoma	Phase 2	Melanoma	October 2004	September 2012	43	NCT00101166

## Data Availability

This is a Review article and there are no new data in this manuscript. Otherwise, all the information is included in the main text.
